# The Effect of Hypothermic Machine Perfusion to Ameliorate Ischemia-Reperfusion Injury in Donor Organs

**DOI:** 10.3389/fimmu.2022.848352

**Published:** 2022-04-29

**Authors:** Laura W. D. Knijff, Cees van Kooten, Rutger J. Ploeg

**Affiliations:** ^1^ Nephrology, Department of Internal Medicine, Leiden University Medical Centre, Leiden, Netherlands; ^2^ Transplant Centre of the Leiden University Medical Centre, Leiden University Medical Centre, Leiden, Netherlands; ^3^ Nuffield Department of Surgical Sciences, University of Oxford, Oxford, United Kingdom

**Keywords:** hypothermic machine perfusion, ischemia-reperfusion injury, HIF, cell death, endothelial dysfunction, immune response

## Abstract

Hypothermic machine perfusion (HMP) has become the new gold standard in clinical donor kidney preservation and a promising novel strategy in higher risk donor livers in several countries. As shown by meta-analysis for the kidney, HMP decreases the risk of delayed graft function (DGF) and improves graft survival. For the liver, HMP immediately prior to transplantation may reduce the chance of early allograft dysfunction (EAD) and reduce ischemic sequelae in the biliary tract. Ischemia-reperfusion injury (IRI), unavoidable during transplantation, can lead to massive cell death and is one of the main causes for DGF, EAD or longer term impact. Molecular mechanisms that are affected in IRI include levels of hypoxia inducible factor (HIF), induction of cell death, endothelial dysfunction and immune responses. In this review we have summarized and discussed mechanisms on how HMP can ameliorate IRI. Better insight into how HMP influences IRI in kidney and liver transplantation may lead to new therapies and improved transplant outcomes.

## Introduction

Several countries use hypothermic machine perfusion (HMP) as standard preservation method for kidney transplantation instead of storing the organ on ice. It has been shown by several clinical studies that HMP is superior to static cold storage (SCS), but the underlying mechanisms are still unknown. Ischemia-reperfusion injury (IRI) is unavoidable during transplantation and is one of the main causes for delayed graft function (DGF) (1). Since HMP is known to affect DGF, it is possible that this positive effect is due to its influence on IRI. To gain a better insight into the effect of HMP on ameliorating IRI. In this review we evaluated the effect of HMP on four main aspects that are involved in the pathophysiology of IRI to gain a better insight into the effect of HMP on ameliorating IRI. This was done by analysing studies comparing static cold storage with HMP.

## History of Machine Perfusion

Since the early beginning of solid organ transplantation, cold preservation has been the gold standard in kidney preservation. Lowering the temperature of the donor organ below 10°C decreases metabolism by approximately 90%, allowing maintenance of donor organ viability and safe preservation. Initially kidneys were cold perfused on a machine, but when better organ preservation solutions were developed, simple SCS in a box with melting ice became the standard in organ preservation due to its simple and effective way to transport the graft. In the past decades, due to the persistent donor kidney shortage, most centres have increasingly been accepting older and higher risk donor kidneys. These kidneys are from donation after circulatory death (DCD) donors or from donors with increased co-morbidity, i.e. hypertension, diabetes or atherosclerotic disease (2). This change of practice often resulted in compromised function and lower graft survival. Also, it became clear that the conventional method of SCS did not suffice for this type of donor organs and improved strategies in preservation appeared to be necessary. This insight resulted in a revisit of continuous machine perfusion, now using novel technologies in medical engineering including oxygenation, both hypo- and normothermic temperatures and modifications of perfusion solutions. Following initial clinical trials demonstrating better outcomes for higher risk donor kidneys (3), several countries have now implemented HMP as the preferred preservation method. In 2009, the first study was published reporting on the results of an international randomized controlled trial with a paired kidney design. One kidney was preserved with HMP whilst for the contralateral kidney SCS was used. In this trial, the method of HMP overall reduced DGF from 26.5% to 20.8% and increased 1 year graft survival from 90% to 94% (4). When analyzing the different subgroups of donor types (ECD, donation after brain death (DBD), DCD), HMP remained superior in decreasing DGF. Subsequent clinical studies and registry reports have been performed and confirmed the beneficial effect of HMP over SCS. Meta-analysis showed that HMP reduced DGF [relative risk (RR) 0.81 with 0.71-0.92 95% confidence interval (CI)] in all deceased donor types in kidney transplantation (DBD fixed-effects analysis with RR 0.84 with 0.69-1.03 95% CI and DCD random-effects analysis with RR of 0.80 with 0.62-1.04 95% CI) (5). A more recent meta-analysis showed a reduced risk of delayed graft function when kidneys were preserved with HMP versus SCS (RR 0.77, 95% CI 0.67-0.90) as well as a trend towards improved graft survival (6). HMP was also shown to be cost effective, because lower DGF and graft failure rates decreased the need for a return to (chronic) dialysis. Beneficial effects of HMP were also shown in a meta-analysis for the liver, by a lower incidence of early allograft dysfunction (EAD), less biliary complications and ischemic cholangiopathy, as well as lower aspartate aminotransferase levels (7). Although short term outcomes in kidney transplantation have improved over the last decades, long term outcomes are still moderate with 70% of DBD and DCD grafts failing between 5 and 10 years posttransplant (8). Two important factors that influence long term outcome in kidney transplantation are the quality of the graft and the immunosuppression administered (9). HMP may potentially alter immunogenicity of the donor organ, thereby improving the quality of the graft. Also, HMP offers a great platform for therapeutic options to potentially reduce the amount of immune suppressive drugs.

## Ischemia-Reperfusion Injury

**Figure 1:**
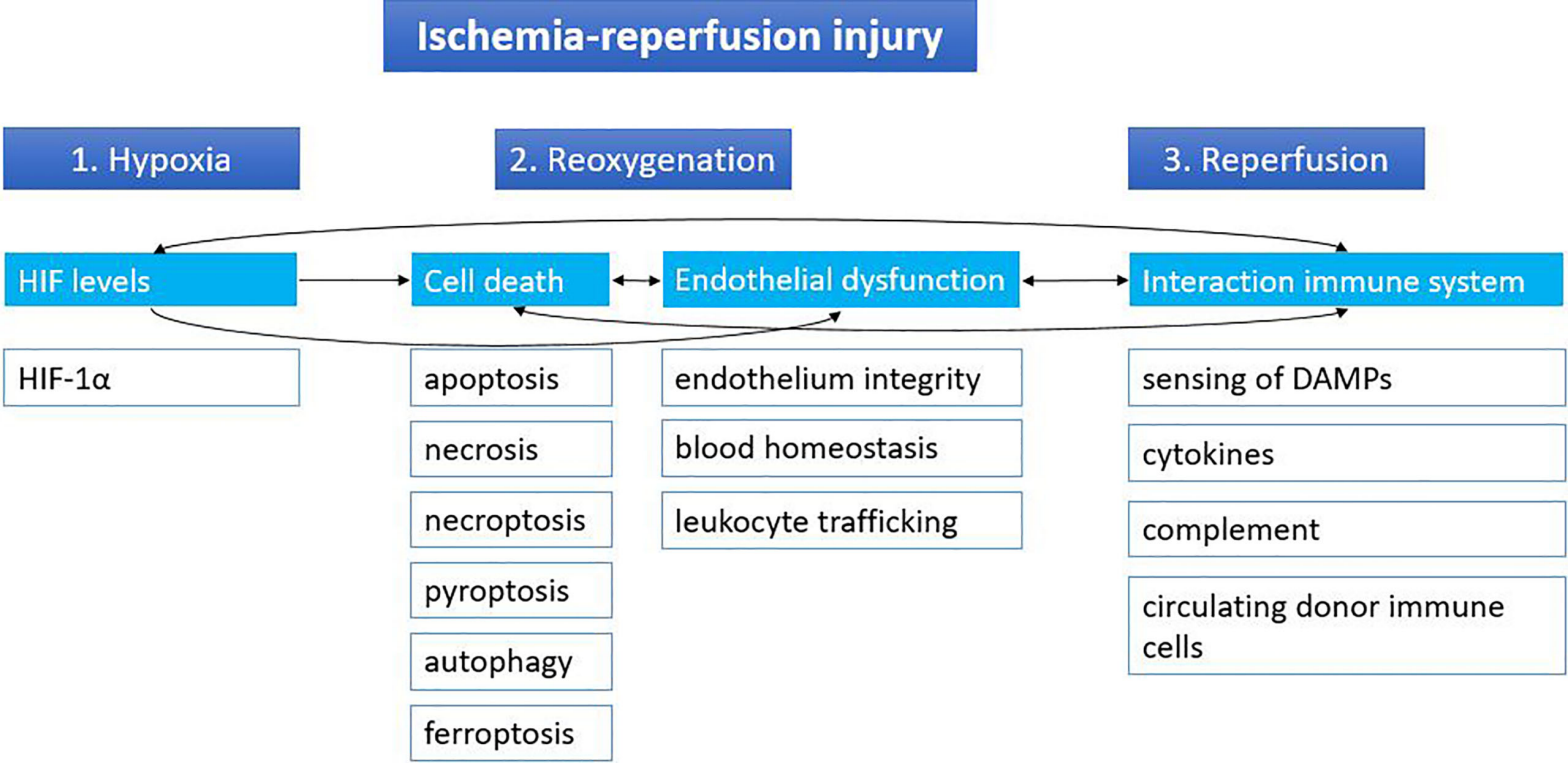
Stages of ischemia-reperfusion injury. Ischemia-reperfusion injury can be divided into three stages based on distinct molecular mechanisms: hypoxia, reoxygenation and reperfusion. Cut-off of the donor organ leads to hypoxia, which leads to changes in HIF levels. During reoxygenation, reactive oxygen species lead to massive cell death and endothelial dysfunction. When the graft is reperfused in the recipient, the immune system of the recipient can react to the graft and its immune cells and vice versa. Arrows indicate the relations between the different mechanisms. DAMPs, damage-associated molecular patterns; HIF, hypoxia inducible factor.

Ischemia followed by reperfusion is inevitable in the context of organ transplantation. The temporary cut-off of the donor organ from the blood supply at the time of procurement until the recipient operation will cause hypoxia, leading to inhibition of the electron transport chain and subsequent lower ATP production. Lower ATP levels will cause a shift toward anaerobic metabolism with dysfunction of sodium-potassium, calcium and sodium-hydrogen pumps. This will result in an imbalance in cellular osmolarity, with as a consequence cell swelling. Anaerobic metabolism also leads to metabolic acidosis from the increased lactic acid levels; decrease of antioxidative agents; and detachment of ribosomes resulting in less protein synthesis. During reperfusion of the graft, there will be a second wave of injury, further damaging the graft, including the release of reactive oxygen species (ROS). The lower levels of antioxidants are unable to neutralize the ROS, leading to cell death. Subsequently, the innate immune system is activated by the profound release of damage-associated molecular patterns (DAMPs) from dying cells. A more detailed review about IRI can be found elsewhere (10). In this review IRI is divided into three main phases: hypoxia, reoxygenation and reperfusion. Although all three phases can be characterized by distinct molecular mechanisms, they are also closely interrelated (**Figure 1**).

## Influence on Hypoxia Inducible Factor Levels

**Figure 2:**
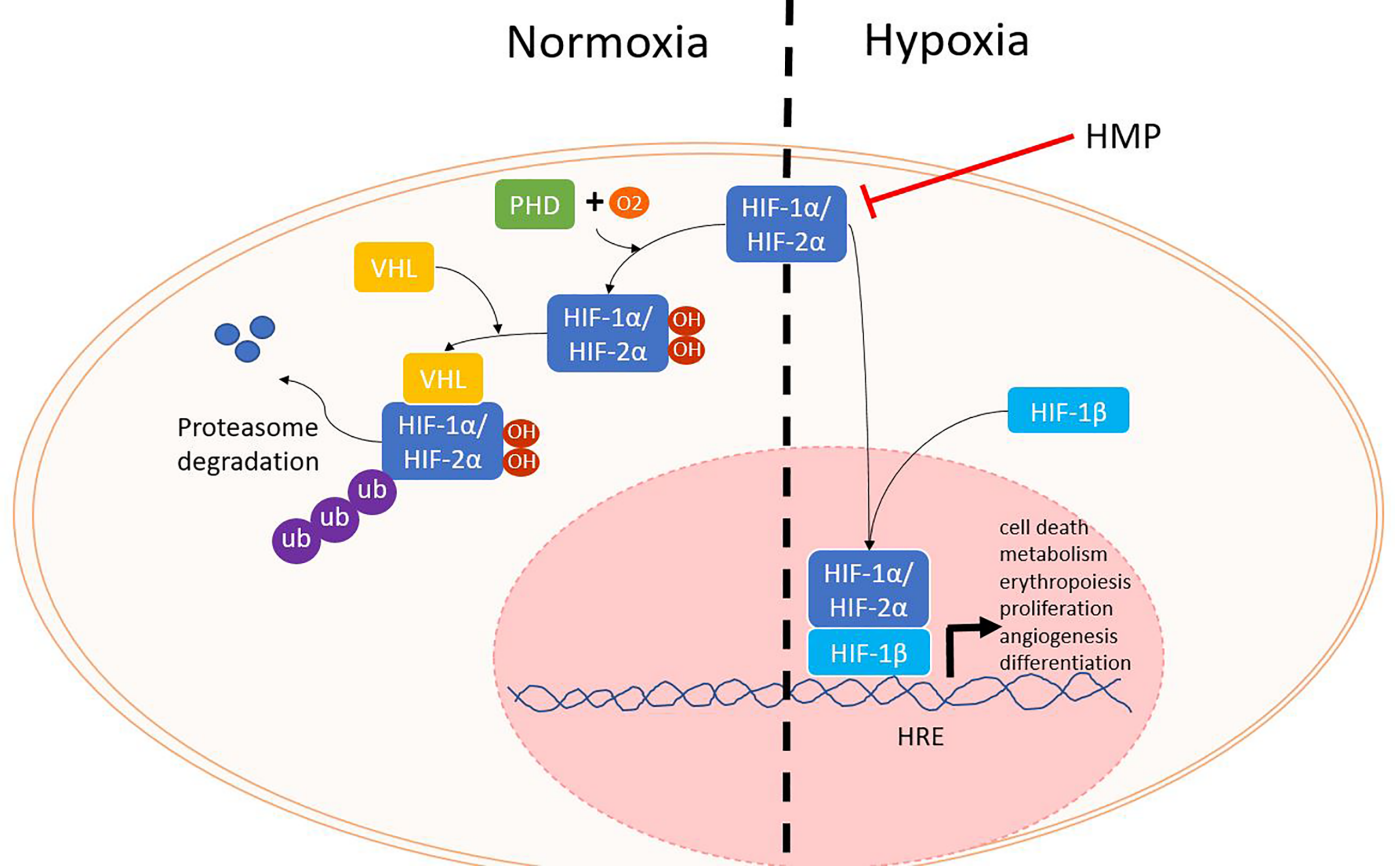
HIF proteins during normoxia and hypoxia. When oxygen levels are sufficient, PHD enzymes use oxygen as a cofactor to hydroxylate HIF-α. Next, VHL will mark HIF-α for degradation by the proteasome. During hypoxia, HIF-α is not degraded and can translocate with HIF-1β to the nucleus where it can bind to HRE and lead to the activation of several different genes. HIF, hypoxia inducible factor; HRE, HIF response element; PHD, prolyl hydroxylase; VHL, von Hippel Lindau.

The first phase of ischemia-reperfusion injury is hypoxia. When oxygen levels are normal, hypoxia inducible factor (HIF) α proteins (HIF-1α, HIF-2α) are rapidly produced, but also degraded. Prolyl hydroxylases (PHD) enzymes use oxygen as a cofactor to mark HIF - α proteins for degradation by the proteasome (**Figure 2**). During hypoxia, PHD enzymes are no longer able to trigger the break down, resulting in increasing HIF-α levels. HIF-α translocates to the nucleus where it binds to the HIF-1β subunit. Together the HIF protein binds to HIF response elements on the DNA, thereby influencing many genes that regulate angiogenesis, metabolism, cell growth and survival (11, 12).

Most studies reported lower levels of HIF proteins (HIF-1α, HIF-2α, HIF-3α and HIF-1β) in HMP-stored donor organs compared with SCS (13–18). Besides lower levels of HIF proteins, improvements on transplant outcome (less damage, better kidney function) were also observed when HMP was applied. However, there is a lot of variability between these studies. Studies were performed with canine, porcine or human organs with HMP duration varying between 3 and 24h. Organs that were subjected to HMP or SCS were liver, limb or kidney. Also, two studies were found where higher levels of HIF-1α and HIF-2α (and downstream factor vascular endothelial growth factor A) were found in HMP perfused human livers. Burlage et al. (19) showed in a human liver model better endothelial cell function with increased HIF-1α levels as measured by Krüppel-like factor 2 (Klf2), endothelial nitric oxide synthase (eNOS), nitric oxide (NO) and thrombomodulin (19). Ito et al. showed in a human liver model that HIF-1α expression increased after HMP (20). They also analysed HIF-1α levels in clinical liver patients and found that higher levels were associated with significantly better graft survival. Unfortunately, to date only an abstract is available of this study, so details about this study are lacking. HIF upregulation that occurs during hypoxia can be explained as a protective mechanism to better handle the ischemic injury (20–23). Studies reporting lower levels of HIF proteins focus more on the hypoxia part, suggesting that lower levels of HIF mean less hypoxia. The two studies reporting higher HIF levels can be compared at best with the studies of Guarrera et al. (17) and Henry et al. (18) that also use a human liver model. Unfortunately, the first study only provides an abstract and does not include the duration of HMP, making it difficult to compare with other studies. The study of Henry et al. provided both clinical outcomes (beneficial for HMP with a shorter hospital stay and a trend towards less EAD and analysis of biopsies and serum (less oxidative stress, inflammatory markers, adhesion molecules and cellular infiltration in the HMP group). Taking along the previously mentioned studies that reported lower levels of HIF, it is likely that HMP reduces the level of HIF proteins despite the great heterogeneity between studies. Nevertheless, these results illustrate the complexity of interpreting data on HIF levels, next to the different molecular consequences of HIF.

As mentioned before, HIF can regulate expression levels of many different genes. Those gene products can have contradictory effects, making it difficult to predict the effect in changing HIF levels. For example, HIF activation can lead to upregulation of both pro- and anti-apoptotic genes. A study by Ravall et al. showed that in a renal cell carcinoma cell line there were opposing effects of HIF-1α and HIF-2α (24). When HIF-1α was expressed, HIF-2α was suppressed and tumour growth was inhibited. When HIF-2α was expressed, HIF-1α was suppressed and tumour growth was increased.. HIF-1α regulates many glycolytic enzymes and is expressed in many different tissues whereas HIF-2α regulates more broadly hypoxia inducible genes like cyclin D, transforming growth factor (TGF)-α and matrix metalloprotease 2 and its expression is more regulated (25, 26). The HIF-3α unit is less studied, but it is thought to inhibit HIF-1α and HIF-2α (26). The expression of the different HIF proteins can vary between different tissues, which could result in different hypoxia responses. In the kidney HIF-1α is expressed in the tubular epithelium, while HIF-2α is expressed in glomerular cells and peritubular interstitial cells (27, 28). HIF-2 has been associated with regulating erythropoietin synthesis (29). Kapitsinou et al. showed in a mouse model that HIF-2α endothelial inactivation resulted in increased expression of cellular infiltration and renal injury markers (27). This was associated with elevated *Vcam1* expression. HIF-2 appears to protect against IRI and could therefore be a potential therapeutic target.

As stated earlier, the different phases of IRI are overlapping. HIF activation has been linked to increased thrombotic factors (30), fibrosis (31) and activating the innate immune cells (32). A rat study showed the effects of changing HIF levels on cell death and inflammation in a hepatic IRI model (33). Overexpression of HIF-1α lead to a protective effect by reducing necrosis, apoptosis, neutrophil infiltration and inflammatory cytokines IL-6 and TNF-α. Inhibition of HIF-1α had the opposite effect and aggravated the IRI injury. These results show the diversity of effects of changing HIF levels, where the protective effect was seen with increasing levels, whereas HMP mainly showed that a decrease lead to better outcomes. Future studies with a paired kidney design, where one kidney is put on HMP while the other is preserved SCS, both with similar cold ischemia times, could provide better insight into changing HIF levels.

## Influence on Cell Death

**Figure 3:**
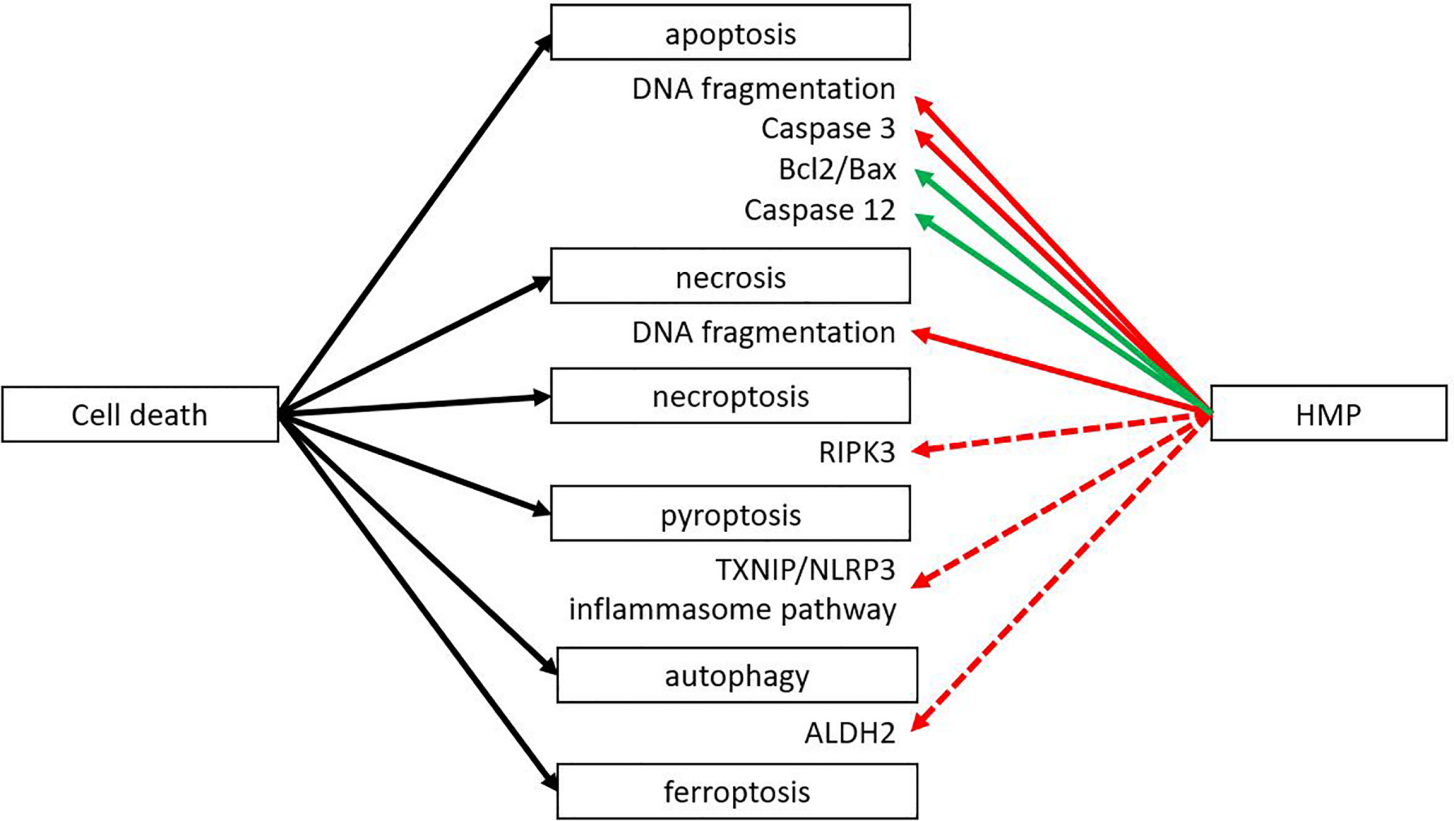
HMP reduces several forms of cell death. Different forms of cell death can occur after ischemia-reperfusion injury. HMP has been found to have an inhibiting effect on apoptosis and necrosis at the end of machine perfusion and after reperfusion in a NMP setting or autotransplantation (red line). The inhibiting effect on apoptosis is *via* inhibition of pro-apoptotic processes and increase of anti-apoptotic factors (green line). There are studies suggesting HMP could also have an inhibiting effect on necroptosis, pyroptosis and autophagy, but more research is needed (red dotted line). The effect of HMP on ferroptosis is yet unclear. ALDH2, aldehyde dehydrogenase; RIPK, receptor-interacting protein kinase; TXNIP/NLRP3, thioredoxin-interacting protein/NOD-like receptor protein.

During the second phase of IRI the reoxygenation leads to the release of ROS, leading to massive cell death. There are different forms of cell death (i.e. necroptosis, pyroptosis, autophagy), but all result in the release of DAMPs that can activate an immune response. However, different forms of cell death could still lead to different DAMPs being released, leading to a different immune response. Apoptosis is generally considered not to activate the immune system. Macrophages that engulf apoptotic cells are stimulated to secrete anti-inflammatory TGF-β and IL-10 (34). Nevertheless, DAMPs can still be released during this form of regulated cell death, as macrophages can also be stimulated to secrete high mobility group box 1 (HMGB1), a well-known DAMP (35, 36). Also, the release during apoptosis of oxidized mitochondrial DNA has been shown to initiate an immune response by activating the nucleotide binding domain and leucine rich repeat (NLR) pyrin domain containing 3 (NLRP3) (37). Necrosis - on the other hand - is also a form of cell death where many DAMPs are released. Necrosis is characterized by organ swelling and membrane rupture, leading to release of the cellular content (10). When comparing the effects of HMP with SCS, the amount of cell death by apoptosis and necrosis was reduced in HMP (**Figure 3**) (38–45). Less cell death most likely translates to less release of DAMPs, leading to less activation of the immune system. Therefore this could contribute to better outcomes with hypothermic machine perfused organs versus static cold stored ones. The ratio of anti-apoptotic Bcl2/pro-apoptotic Bax increased when organs were perfused with HMP versus SCS (41, 42). A higher Bcl2/Bax ratio will prevent the occurrence of apoptosis (46). Apoptosis is initiated from an extrinsic pathway that activates caspase 8 and 10 or *via* an intrinsic pathway activating caspase 9. Once activated, the cascade of caspases leads to a regulated dismantling of the cell. Caspases also play a role in inflammation by recognizing bacterial products such as lipopolysaccharide, leading to pyroptosis and activating pro-inflammatory cytokines by cleavage. It has been suggested that caspase 4 and 5 can directly recognize lipopolysaccharide in the cytosol (47). Caspase 3 has been found to be downregulated after reperfusion while caspase 12 was found upregulated when organs were perfused with HMP versus SCS (18, 38–40, 42, 48–51). Caspase 3 is an essential effector caspase that plays a role in both the intrinsic and extrinsic pathway. Caspase 12, on the contrary, is known as a negative regulator of inflammation by inhibition of caspase 1, which is responsible for cleavage of pro-IL-1β and IL-18 to their active form (47, 52).

Most research about the effect of HMP on cell death is focused on apoptosis and necrosis. Other forms of cell death such as necroptosis, pyroptosis or autophagy have not yet been studied extensively, although it is known that different forms of cell death may activate different immune pathways. Necroptosis has been associated with IRI in liver transplantation (53), ischemic brain injury (54) and renal IRI (55, 56). A study performed in rabbit kidneys showed that the expression of receptor-interacting protein kinase 3 (RIPK3) was significantly lower in the HMP group versus the SCS group both at the protein and mRNA level (51). The RIPK1-RIPK3 complex is an important regulator of necroptosis by forming the necroptosis-inducing complex called the necrosome (57).

The influence of HMP on pyroptosis has been investigated in liver perfusion in a rat study (58). The investigators studied the effect of hypothermic oxygenated machine perfusion (HOPE) following a period of SCS on the inflammasome pathway that plays a role in pyroptosis. The HOPE group showed less pyroptosis, likely by blocking the interaction between thioredoxin-interacting protein and NLRP3. The role of decreasing pyroptosis on transplant outcome was studied by Noda et al. in lung perfusion. They showed that lung perfusion of rat heart-lung blocks showed significantly better lung function and lower IL-6 levels when perfused with a leukocyte filter (59). Leukocytes trapped in the filter were analysed for cell death and they found that 26% of the cells were pyroptotic (caspase 1 positive, 7AAD negative), 16% apoptotic (Annexin V positive, 7AAD negative) and 40% necrotic (Annexin V positive, 7AAD positive). Administering a caspase-1 inhibitor during perfusion to inhibit pyroptosis showed better lung function and lower mRNA levels of proinflammatory cytokines IL-6, TNF-α and IL-1β, comparable with perfusion with a leukocyte filter. A limitation of this study is that only mRNA levels of cytokines were measured instead of protein levels. Caspase 1 plays an important role in activation and secretion of IL-1β and has also been linked to TNF-α and IL-6 secretion from macrophages (60).Therefore, it may be of interest to study whether the effects of blocking pyroptosis are similar on the protein level as found to be on the mRNA level.

The role of autophagy during IRI has also been studied in various organs (61–63). According to van Erp et al. the degree of autophagy in the donor can also be influenced by age and gender (64). During the first ischemic phase, it is proposed that autophagy acts as a protection mechanism whereas during the reperfusion stage excessive autophagy results in cell death (65). A study in rabbits showed higher levels of phosphorylated aldehyde dehydrogenase 2 (ALDH2) in HMP-perfused kidneys versus SCS (63). ALDH2 influences autophagy *via* expression of 4-HNE that regulates the Akt/mTOR autophagy pathway, suggesting that under HMP conditions, autophagy increases when compared to SCS. Administering an ALDH2 agonist to enhance autophagy resulted in (i) better kidney function as shown by lower serum creatinine, (ii) lower oxidative stress levels measured by malonaldehyde and superoxide dismutase 2, and (iii) better inflammatory profile as demonstrated by lower levels of TNF-α, IL-6 and higher levels of IL-10. Administering an antagonist, to decrease autophagy, lead to the opposite results. A study by Zeng et al. looked into the role of HOPE in upregulating autophagy to alleviate liver IRI in a rat model (44). HOPE increased expression of autophagy-related proteins and was associated with better liver function as measured by alanine transaminase, aspartate transaminase and lactate dehydrogenase compared with SCS. Administration of the autophagy inhibitor 3-MA attenuated the protective effect. Perfusion with 100% nitrogen showed similar results as under SCS conditions, indicating that the effect of HOPE treatment was not due to a washout during perfusion. It also demonstrated that a minimal oxygen level is required for the protective effect of HMP. According to Boteon et al. autophagy is important for removal of harmful substances and providing energy during cell stress (66). Normothermic machine perfusion (NMP) has been suggested to increase autophagy by maintaining normal calcium levels and by providing shear stress. Although more research is needed to evaluate the effect of HMP on autophagy, it is likely similar to NMP. During HMP the perfusate contains calcium and there are also low levels of shear stress detectable.

Another form of cell death is ferroptosis, which is iron-dependent. Excessive amounts of iron can lead to the generation of ROS through the Fenton reaction (67). Several studies showed that ferroptosis could play a role in IRI in the liver and kidney (68–70). Ferroptosis was also shown in several cell lines exposed to continuous cold stress, therefore it could be interesting to look into the role of ferroptosis during machine perfusion at subnormothermic (25-35°C) or normothermic (37°C) temperatures as well (71). Currently, no studies have looked into the role of HMP on ferroptosis.

Most studies use a TUNEL (Terminal deoxynucleotidyl transferase dUTP nick end labelling) assay to measure apoptosis *via* detection of DNA fragmentation. However, this assay can also detect necrosis, pyroptosis and possibly other forms of cell death (72–74). Knowing which forms of cell death are affected and which are not, is also important to identify specific targets to reduce IRI. A combination of multiple forms of cell death could also be possible. Recently, a new protein complex was identified as the PANoptosome (75). It drives the three main forms of programmed cell death, namely pyroptosis, apoptosis and necroptosis. The PANoptosome contains RIPK1, caspase 8, NLRP3 and apoptosis-associated speck-like protein (ASC) containing a caspase recruitment domain. Therefore, it contains molecules that are critical for programmed cell death. The different pathways can be activated together or separately and crosstalk between pathways occurs (75, 76).

In conclusion, it appears that HMP reduces the amount of cell death by decreasing apoptosis and necrosis. It is possible that HMP also reduces other forms of cell death like necroptosis, autophagy, pyroptosis and ferroptosis, but so far not many studies have looked into this. The effect of HMP on cell death will likely affect other mechanisms as well. Cell death plays an important role in activating the innate immune system *via* the release of DAMPs and caspases are critical in cleaving several cytokines into their active form. If cell death occurs in endothelial cells, this will disrupt the glycocalyx.

## Influence on Endothelial Dysfunction

**Figure 4:**
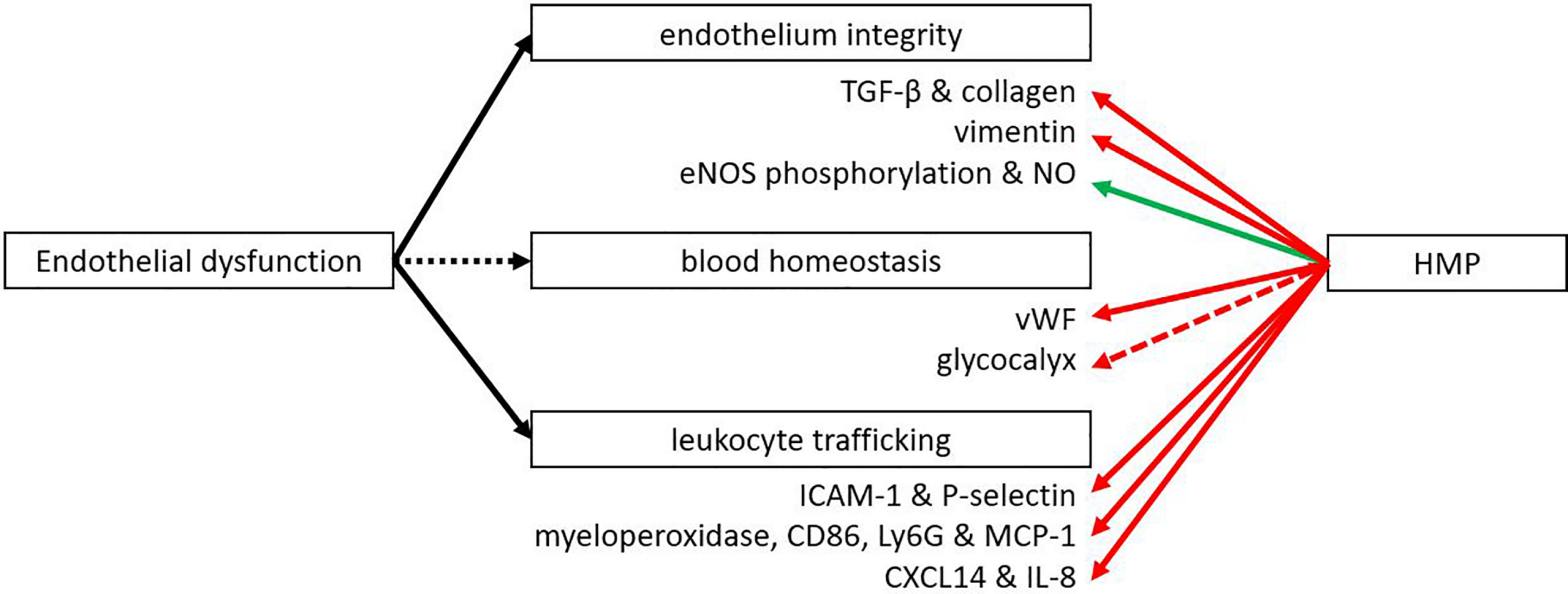
HMP influences several functions of the endothelium. After HMP or reperfusion (NMP or autotransplantation) there is less leukocyte trafficking compared to SCS treated organs (red line). Also less thrombotic factors, fibrosis and EMT were observed. However, the vascular tone was improved when organs were treated with HMP instead of SCS (green line). More studies are needed to confirm the effect of HMP on the glycocalyx (red dotted line). eNOS, endothelial nitric oxide synthase; ICAM, intercellular adhesion molecule; Ly6G, lymphocyte antigen 6 complex locus G6D; MCP-1, monocyte chemoattractant protein-1; NO, nitric oxide; TGF-β, transforming growth factor β; vWF, von Willebrand factor.

The endothelium plays a major role in inflammation with leukocyte adhesion and vascular health. Multiple studies have shown that IRI leads to the upregulation of adhesion molecules like E-cadherin and intercellular adhesion molecule 1 (ICAM-1) that enable leukocyte adhesion and neutrophil infiltration (77–79). Following HMP and reperfusion, many studies showed better endothelial function as measured by less transmigration of immune cells (**Figure 4**). In addition, less fibrosis after kidney preservation was measured by Sirius Red staining as well as by lower levels of pro-fibrotic transforming growth factor β (39, 80–82). A porcine model was used to study the effects of HMP on fibrosis, with the exception of Liu et al. (82) who used a rabbit model. Fibrosis is significantly associated with chronic graft dysfunction (11). Endothelial dysfunction has been mentioned as an important initiator and maintainer of fibrosis (83, 84). Therefore, we suggest that the level of fibrosis may indicate endothelial dysfunction. P-selectin is important for the rolling of leukocytes to ultimately invade the tissue and ICAM-1 is important for adhesion of leukocytes to endothelial cells. Lower expression of P-selectin and ICAM-1 were observed when HMP was applied versus SCS (13, 17, 18, 43, 49, 85). Reduced leukocyte rolling and adhesion with HMP was shown in liver models in rat (49), mouse (43), dog (13) and human (17, 18, 85). Most studies showed lower expression of P-selectin and ICAM-1 after perfusion (13, 17, 18, 85) or reperfusion (13, 43, 49), while Henry et al. (18) also showed a decrease in P-selectin levels after transplantation. Less cellular infiltration was also shown by a reduction in invading neutrophils and monocytes as measured by myeloperoxidase, CD68, Ly6G and reduced levels of monocyte chemoattractant protein-1 (18, 43, 80, 86, 87). A reduction in cellular infiltration was seen across various organisms (mouse, rabbit, human, pig) after perfusion, in reperfusion models and after transplantation. This was supported by a decrease in chemokines CXCL14 and IL-8 as shown after perfusion in a canine or human liver (13, 18, 85). Henry et al. (18) showed both after perfusion and transplantation an downregulation of CXCL14 and IL-8. Endothelial-to-mesenchymal transition (EMT) was observed less frequently as measured by lower levels of EMT marker vimentin after perfusion of porcine kidneys (80, 81). EMT is an important factor that contributes to fibrosis and chronic graft failure (88). A downregulation of the thrombotic von Willebrand-factor was also observed after reperfusion in liver transplantation in mice or pigs (44, 45). The vascular tone of the endothelium is important for sufficient flow. Vasodilation can be regulated by eNOS phosphorylation, leading to an increase in NO levels, which were found to be upregulated after reperfusion of HMP-treated organs (19, 89–91). This was true for both kidneys and livers and in different organisms (pig, rat, human). For the porcine kidney transplantation, higher levels of eNOS were also found after transplantation (90). In conclusion, a beneficial effect of HMP on endothelial integrity and function has been demonstrated at different levels.

Many of these beneficial effects on the endothelium are most likely caused by the activation of Klf2, which gets activated upon flow-mediated shear stress. Besides regulation by flow, Klf2 can also be negatively regulated by proinflammatory cytokines such as TNF-α and H2O2 resulting from oxidative stress (92). Klf2 can regulate many different processes in the endothelium like angiogenesis, vascular tone, thrombosis, inflammation, immune regulation and oxidative stress. It is known to downregulate HIF-1α, vascular endothelial growth factor, endothelin-1, vascular cell adhesion molecule and E-selectin, while thrombomodulin and eNOS are some of the upregulated markers (92, 93). It has already been shown by multiple studies that Klf2 gets upregulated during HMP by the shear stress created from the pump (82, 94, 95). The upregulation of eNOS phosphorylation is important for NO levels. NO is an important vasodilator, allowing for sufficient blood flow through the organ. Besides regulating vascular tone, NO also plays a role in endothelial cell migration, proliferation, angiogenesis and it has anti-inflammatory properties by inhibiting leukocyte adherence (96).

When discussing the endothelium, another essential factor is the glycocalyx, a thin layer consisting of proteoglycans that covers the endothelium. This cover is important for leukocyte and platelet adhesion, coagulation and transferring shear stress to endothelial cells (97). The glycocalyx is also an important place where several enzymatic reactions take place due to the docking function of the glycosaminoglycans. Damage to the glycocalyx is a direct consequence of IRI, as demonstrated in several studies (98–100). A large part of the damage to the glycocalyx happens during the reperfusion phase (101). One study looked in more detail into the glycocalyx degradation during human liver transplantation and found that syndecan-1, a biomarker of glycocalyx degradation, was released during reperfusion (102). However, heparan-sulphate levels, another biomarker for glycocalyx degradation, were lower in effluent veins compared with portal venous blood, suggesting binding or uptake of heparan-sulphates. This uptake might suggest repair of the damaged graft. This is supported by a study in kidney transplantation, where the thickness of the glycocalyx increased in time after reperfusion (103). Therefore, it could be beneficial to perfuse donor grafts for a longer period of time to give the glycocalyx time to repair itself, although more research is needed to confirm this. One study in a porcine model of brain death showed that HMP could potentially be used as a platform to restore the glycocalyx by infusion of corline heparin conjugate, heparin molecules that strongly bind to tissue with heparin affinity. By labelling the heparin molecules, binding to the damaged endothelium could be demonstrated (104). Unfortunately, no results on function or outcome were reported.

All in all, there appear to be many functions of the endothelium that are influenced by HMP. Most studies focussed on the infiltration of immune cells and fibrosis and noticed a reduction when using HMP versus SCS. Endothelial dysfunction also affects other mechanisms of IRI. ICAM-1 has also been studied in cancer cells, where they found that an upregulation of ICAM-1 increased cell survival (105). For P-selectin it was found that inhibition of P-selectin reduced apoptosis in endotoxin-induced liver injury in a mouse model (106). Besides leukocyte recruitment, activation of the coagulation system can also lead to an immune response. Components of the clotting system like fibrin can enhance adherence of immune cells and facilitate migration (107). Cross-talk between the complement system and the coagulation system can further activate the immune system (108).

## Influence on Innate Immune Response

**Figure 5:**
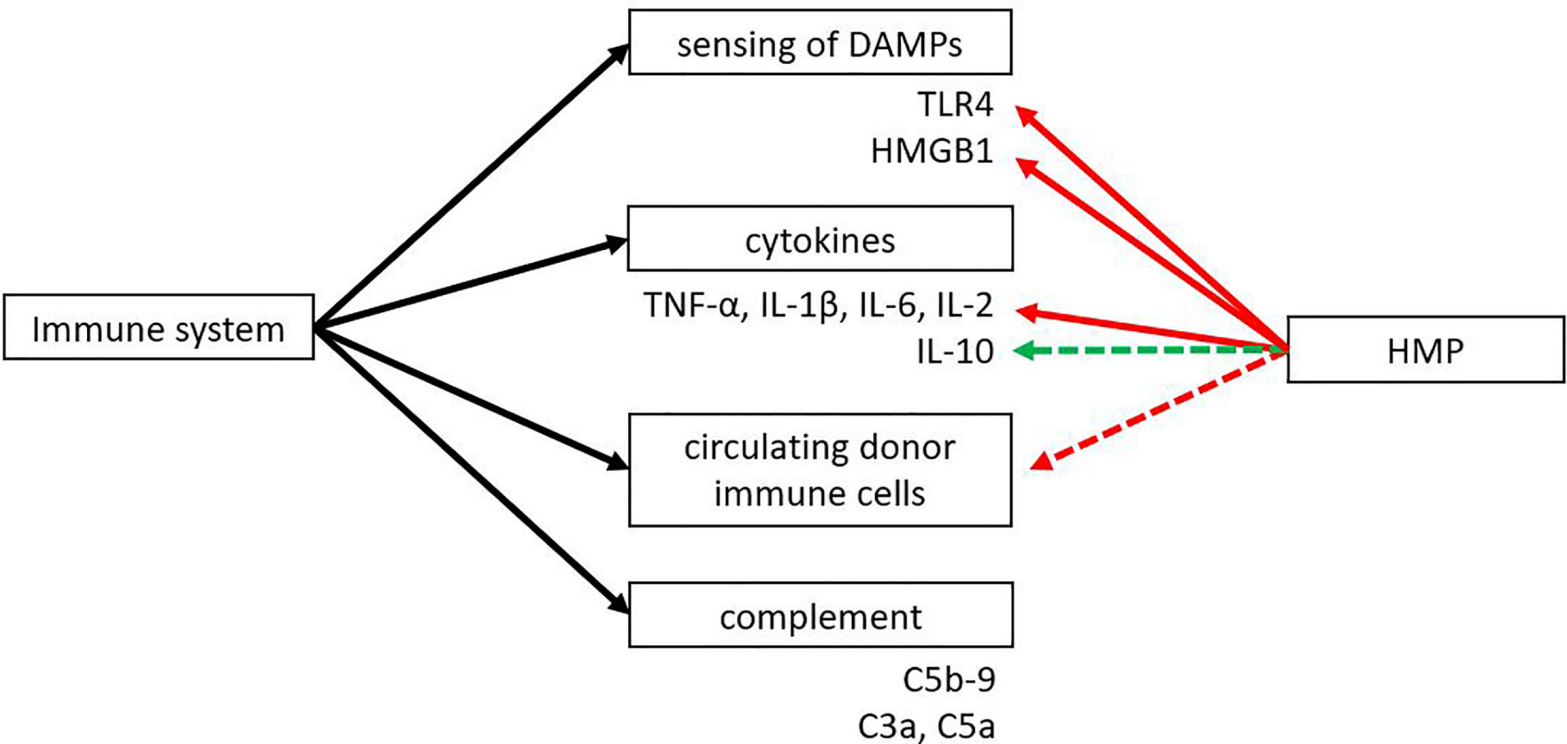
The effect of HMP on dampening the immune response. HMP has shown to reduce sensing of DAMPs and pro-inflammatory cytokines compared to SCS (red line). Some studies suggest there might be an upregulation of anti-inflammatory cytokines and a reduction in circulating donor immune cells (green and red dotted line). The effect of HMP on the complement system is still unknown. DAMPs, damage-associated molecular patterns; HMGB1, high-mobility group box 1; TLR, Toll-like receptor.

Both the innate and adaptive immune system play an important role in transplantation. An eligible donor-recipient match has to be found, immunosuppressive drugs have to be taken daily and there is the risk of allograft rejection of the donor organ. The innate immune system plays an important role in IRI. Toll like receptor 4 (TLR4), one of the best characterised TLRs, is able to sense DAMPs that are released during IRI. HMBG1 is one of the most described DAMPs and it has been found to play a role in IRI by activating TLR4 (109). HMGB1 normally resides within the nucleus, where it plays a role in transcription and chromatin modelling (110). It can be actively released by immune cells like dendritic cells or macrophages or passively released upon cell death. Extracellular HMGB1 can bind to TLR4, TLR2 or the receptor for advanced glycation end-products (RAGE) to promote inflammation (110, 111). Several TLRs have been found upregulated in ischemia, mostly TLR2 and TLR4. Activation of TLR4 leads to increased expression of proinflammatory cytokines and adhesion molecules, attraction of neutrophils and macrophages, and activation of circulating immune cells (11, 112–114). Several studies have shown that TLR4 expression strongly correlates with renal graft dysfunction in rats and that TLR4 knockout mice are protected against IRI (14). Both TLR4 and HMGB1 were found to be downregulated when comparing HMP versus SCS (**Figure 5**) (41, 44, 45, 51). Besides TLR4 and HMGB1, pro-inflammatory cytokines TNF-α, IL-1β, IL-6 and IL-2 were reported to be downregulated after HMP treatment (17, 18, 42, 43, 45, 51, 85, 86, 91). The possibility of further reducing pro-inflammatory cytokine levels during HMP was shown in a study that compared porcine kidneys in a reperfusion model with or without a cytokine filter (115). Kidneys that were perfused with a cytokine filter showed lower levels of IL-6 and IL-8 and higher blood flow. No effect on kidney function based on creatinine clearance was found, but it might be that more processes play a role in improving kidney function. For instance, a cytokine filter is non-specific which means that anti-inflammatory cytokines are removed as well. A shortcoming of most studies reporting on changes in the immune system after HMP is that many only look at pro-inflammatory cytokines while anti-inflammatory cytokines like IL-10 are not as often investigated.

Next to the release of DAMPs or inflammatory mediators, molecular alterations in the donor organ following IRI can also result in the activation of innate immunity *via* the complement system. The complement system can be activated *via* the classical, lectin or alternative pathway. Activation of the complement system by DAMPs released from IRI can lead to the cleavage of C3 and C5 and the formation of the membrane attack complex. Cleavage products C3a and C5a can act as anaphylatoxins to activate immune cells (116, 117). A complexity in complement research is that activation of the complement system can vary between organs. IRI in the liver leads to activation of the classical pathway, while IRI in the heart activates the classical and lectin pathway (117). Therefore, more studies should look into the effect of hypothermic machine perfusion on complement activation.

Probably due to the short time of perfusion and the cold temperature not many studies have looked into the effect of HMP on the adaptive immune system. Several studies have looked into the circulating cell types during machine perfusion and found large amounts of immune cells with flow cytometry (118, 119). Although the consequence of circulating immune cells during machine perfusion is not clear yet, it could have a beneficial effect by immunodepletion of the graft.

Just like the other mechanisms, immune activation also influences the previous discussed mechanisms involved in IRI. Jantsch et al. (120) showed that TLR activation on mouse dendritic cells can also lead to stabilization of HIF-1α under normoxic conditions. This increase in HIF-1α resulted in the transcription of inflammatory target genes *Ptgs2* and *Nos2*, whereas increase of HIF-1α by hypoxia lead to increased transcription of HIF-1α target genes *Glut1* and *Pgk1*. Besides regulation by PHD, pro-inflammatory cytokines have also been reported to regulate HIF-1α (121). TNF-α and IL-1β have been proposed to increase HIF-1α levels *via* various mechanisms at pre- and posttranscriptional levels. However, most information is obtained from cell studies, so further investigations in transplantation models are needed to confirm this. Immune activation can also lead to cell death and endothelial dysfunction. Activation of the complement system leading to the membrane attack complex can induce cell death *via* lysis (117). In an IRI mouse model it was also shown that activation of the terminal complement pathway lead to shedding of the glycocalyx, indicated by accumulation of glycocalyx components syndecan-1, hyaluronan and heparan sulphate (122).

## Interventions During Hypothermic Machine Perfusion

Machine perfusion provides an ideal platform to use for interventions. Current interventions that are being exploited are RNA silencing, stem cell therapy and complement blockade (123). One study looked into the use of lentiviral vectors encoding short hairpin RNAs that target the β2-microglobulin of the major histocompatibility complex 1 during sub-normothermic *ex vivo* rat kidney perfusion (124). They found decreased transcription levels of β2-microglobulin and pro-inflammatory cytokine levels, while increased levels of anti-inflammatory cytokines were found. Genetic modification showed no additional cell death, showing feasibility of this technique.

Mesenchymal stromal cells (MSCs) are also an emerging topic in this field. Due to the immune modulating properties of MSCs both the innate and adaptive immune response could be controlled. MSCs could also be beneficial for tissue repair (125). The TRITON study was a single centre randomized prospective study where MSCs were infused 6 and 7 weeks after renal transplantation in combination with reduced immunosuppressive drugs. It showed that MSC therapy was safe and feasible and also showed higher numbers of regulatory T cells in peripheral blood (126). Due to the size of the MSCs they will get stuck in the lung capillaries when given to the recipient systemically *via* the blood stream. Therefore, *ex vivo* machine perfusion could be an attractive way to give MSC therapy. In a renal porcine autotransplantation model it was shown by the MePEP consortium that giving MSC therapy during NMP was safe and feasible and MSCs ended up in the renal cortex (127). However, in the short follow-up time of 14 days no beneficial effects on function could be shown despite prolonged warm and cold ischemia times. Thompson et al. (128) added multipotent adult progenitor cells added during NMP of discarded human kidneys. They showed that this therapy may increase urine production and microvascular perfusion, upregulate anti-inflammatory cytokines and downregulate injury markers and pro-inflammatory cytokines (128).

Activation of the complement system already occurs early-on in deceased donors as shown by Damman et al., as elevated C5b-9 levels were found in the plasma of deceased donors (129). The elevated levels were also associated with biopsy-proven acute rejection. A phase 1 study where complement is inhibited using C1-INH is being conducted to try to decrease systemic inflammation and DGF incidence in expanded criteria donors as a donor pre-treatment strategy (NCT02435732). Complement inhibitor C1-INH is a serine protease that can regulate the classical, lectin and alternative pathway (130). Machine perfusion could provide an ideal environment to target the complement system specifically in the graft instead of the whole body. The EMPIRIKAL trial was the first study to look into the administration of complement inhibitor Mirococept at time of transplantation to prevent DGF (ISRCTN49958194) (131, 132). The inhibitor is given during a 15min flush of the kidney while the organ is on ice slush. Mirococept is designed to inhibit the complement system at the C3 level. Because the first dose of 10mg did not show a significant difference from the control, the study was stopped to conduct a dose study first. A dose finding study was initiated in normothermic machine perfused porcine kidneys showing that 80mg of Mirococept was the optimal dose (132). It showed to be safe as minimal washout into the circulation occurred and no detrimental effects on flow parameters or histology were observed, opening the path to further clinical development. In this study the inhibitor was not given during HMP, but during a short flush. It would be interesting to study if Mirococept, when given during HMP where would be circulating through the organ for several hours, could be administered in a lower dose.

## New Areas of Interest

With HMP being implemented and clinically used in kidney and liver preservation in several countries, research into machine perfusion has emerged. Due to novel technology, nowadays also *ex-vivo* NMP has become feasible, although its clinical implementation is still in its infancy. The potential advantages of NMP over HMP are its ability to restore cellular function, upregulate protective repair mechanisms and allow better assessment of function. At the same time it provides a platform for cell therapy i.e. administration of mesenchymal stem cells (133). The first clinical trial performed by Hosgood and Nicholson et al. showed that 1-hour normothermic perfusion of human donor kidneys with subsequent transplantation is safe and feasible (134). Due to better assessment of human DCD kidneys, that were declined for transplantation, this group was able to reverse that decision and successfully transplant several kidneys, thereby increasing the donor pool.

A topic of interest in current HMP research is the presumed positive effect of addition of oxygen during cold machine perfusion. Due to the low temperature (approximately 10°C), the metabolism of the graft is reduced by 90%. Initially, it was thought that addition of oxygen was not necessary at that level of reduced metabolism and might actually increase detrimental ROS formation. New insight obtained by pre-clinical work in kidney and liver (135–137) showed improved recovery of transplanted organs when oxygenated perfusion had been used. Recently, the COMPARE trial by the COPE consortium showed in a multicentre clinical context that oxygenated HMP of older DCD donor kidneys was better than non-oxygenated HMP in terms of kidney function, graft survival and rejection rate (ISRCTN32967929) (138). The POMP trial of the COPE consortium compared in higher-risk ECD kidneys conventional SCS to a preservation of first SCS, then followed by a brief period of oxygenated HMP. This large clinical study did not detect any difference in function or survival. This suggests that a brief period of 4.5h oxygenated HMP at the end of preservation is not sufficient for oxygenated HMP to have beneficial effects. It might be best to start oxygenated HMP as soon as possible after organ procurement (139). To date, several clinical trials investigating hypothermic oxygenated machine perfusion in liver and kidney transplantation are ongoing.

As in many other fields, studies looking into machine perfusion are still mainly performed in animal models. Choosing the right animal model however is of importance to be able to translate the newfound knowledge to the human situation. Lerink et al. showed that there is still a big translational gap in many preclinical IRI models (140). This was also shown in the above mentioned EMPERIKAL trial that was first tested in a rat model. Based on those results, the dose range for humans was decided. However, when tested in the pig it was shown that the human dose should have been 12 times higher than estimated from the rat study (132). The porcine transplantation model appears to be the best simulation of human conditions due to similar physiology, size and immune system. Several groups have started to use slaughterhouse pig organs to test various aspects of organ perfusion which reduces the need for animal house pigs n the early exploratory stages (141). In a next step, including experimental transplantation, animal house models will be required before phase 1 studies in humans can be ethically justified.

## Summary

In conclusion, machine perfusion and in particular HMP appears to influence many different pathways involved in IRI. The goal of this review was to compare HMP with the old gold standard SCS. Studies are now available including clinical evidence that HMP has beneficial effects on outcomes such as immediate function and survival but also on important mechanisms that are involved in IRI: HIF levels, cell death, endothelial dysfunction and the innate immune response. To obtain better insight in the mechanisms of injury and repair, future studies should focus on analysis of the effects of HMP on all four mechanisms. This will allow the discovery of underlying relationships and clinically relevant pathways. It will also lead to the development of targeted interventions to increase viability whilst possibly modulating the graft and rendering it less immunogenic. This may help to reach the goal to enhance function and prolong survival avoiding chronic graft dysfunction after 5-10 years due to progressive scarring of the transplanted organ.

## Author Contributions

LK, CK, and RP came up with the conception and design. LK wrote the manuscript and CK and RP revised the manuscript. All authors have read and agreed to the published version of the manuscript.

## Conflict of Interest

The authors declare that the research was conducted in the absence of any commercial or financial relationships that could be construed as a potential conflict of interest.

## Publisher’s Note

All claims expressed in this article are solely those of the authors and do not necessarily represent those of their affiliated organizations, or those of the publisher, the editors and the reviewers. Any product that may be evaluated in this article, or claim that may be made by its manufacturer, is not guaranteed or endorsed by the publisher.
